# The Impact of Modern Therapeutic Methods on the Oxidant-Antioxidant Equilibrium and Activities of Selected Lysosomal Enzymes and Serine Protease Inhibitor in Amateur Athletes

**DOI:** 10.1155/2020/9792836

**Published:** 2020-08-20

**Authors:** Łukasz Sielski, Paweł Sutkowy, Katarzyna Pawlak-Osińska, Alina Woźniak, Agnieszka Skopowska, Bartosz Woźniak, Jolanta Czuczejko

**Affiliations:** ^1^Department of Pathophysiology of Hearing and Balance, Ludwik Rydygier Collegium Medicum in Bydgoszcz, Nicolaus Copernicus University, Toruń, Poland; ^2^Multidisciplinary City Hospital, Rehabilitation Center, Bydgoszcz, Poland; ^3^Department of Medical Biology and Biochemistry, Ludwik Rydygier Collegium Medicum in Bydgoszcz, Nicolaus Copernicus University, Toruń, Poland; ^4^Department of Laser Therapy and Physiotherapy, Ludwik Rydygier Collegium Medicum in Bydgoszcz, Nicolaus Copernicus University, Toruń, Poland; ^5^Department of Neurosurgery, Stanisław Staszic Specialist Hospital, Piła, Poland; ^6^Department of Psychiatry, Ludwik Rydygier Collegium Medicum in Bydgoszcz, Nicolaus Copernicus University, Toruń, Poland

## Abstract

Deep electromagnetic stimulation (DEMS) and low-frequency ultrasound (US) are new physical therapy methods used in the rehabilitation of the musculoskeletal system and wound healing. They are applied locally to treat the injured tissues. The beneficial effects of these methods in supportive care have been documented, but accurate biochemical effects are not known. The goal was to assess the effect of single DEMS and US sessions on the oxidant-antioxidant equilibrium, as well as the activities of lysosomal hydrolases and *α*_1_-antitrypsin (AAT) in peripheral blood of juvenile injured amateur athletes. In the athletes with low back pain (DEMS treated, *N* = 16) and pain in the shoulder or ankle joint (US treated, *N* = 14), as well as in healthy control amateur athletes (DEMS treated, *N* = 14; US treated, *N* = 17), before the sessions and 30 minutes and 24 hours after them, the levels of the following parameters were determined: thiobarbituric acid reactive substances (TBARS) in erythrocytes and plasma, superoxide dismutase (SOD), glutathione peroxidase (GPx), and catalase (CAT) in erythrocytes, as well as acid phosphatase (AcP), arylsulfatase (ASA), cathepsin D (CTS D), and *α*_1_-antitrypsin (AAT) in serum. After both procedures, the levels of parameters changed in a negligible manner, excluding the cathepsin D activity, which was statistically significantly lower 30 min and 24 h after US in the control athletes compared to the baseline activity determined directly before the procedure (47.5% and 55.7% differences, respectively). Similar tendency was observed after DEMS (*p* > 0.05). The procedures, especially low-frequency US, decrease lysosomal proteolytic activity and do not significantly disrupt the oxidant-antioxidant and lysosomal equilibriums in the peripheral blood both of healthy and injured athletes. No systemic acute-phase response of AAT was also detected in the athletes after both procedures. This trial is registered with CTRI/2018/01/011344.

## 1. Introduction

Technical advances in the development of medical devices have a significant effect on the improvement in treatment of sports injuries, diseases, and wounds characterized by acute or chronic pain and inflammation. The new methods allow to limit the amount of the administered medicines. Such methods are deep electromagnetic stimulation (DEMS) and low-frequency ultrasound (US). DEMS is used in order to alleviate pain in the treatment of musculoskeletal disorders and spine problems, as well as in diseases such as rheumatoid arthritis (no reports on inflammation and other phenomena). The method involves variable/pulsed magnetic field with the induction of up to 2.5 Tesla (T) in the frequency range from 1 to 50 Hertz (Hz). Tissues are stimulated locally to the penetration depth of the field, i.e., approx. 10 cm, passing through clothing and tissues, including bones [1–3]. Low-frequency US has similar depth of action but is used with a gel (neutral for health; direct contact of device with the patient's skin) in continuous or pulsed mode. Traditional therapeutic US uses high frequencies, typically ranging between 1 and 3 megahertz (MHz). However, low-frequency US (22–40 kilohertz, kHz) was shown to be more effective (deeper impact and better results). Ultrasound induces similar results to DEMS, but is much better examined. US triggers thermal (an increase of temperature), if applied in a continuous manner, and nonthermal effects (acoustic cavitation and microstreaming) [4]. Low-frequency US in pulsed mode stimulates fracture healing, probably promoting proliferation and differentiation of chondrocytes, which is mediated by TGF-*β*1 [5]. In turn, the effects of ultrasound on wound healing, chronic ulcers, fracture healing, and osteoradionecrosis may be explained by the enhancement of angiogenesis mediated by IL-8, bFGF, and VEGF [6]. Chronic, difficult to heal wounds (e.g., pressure ulcers, surgical wounds, and diabetic foot ulcers) are especially effectively treated using low-frequency US without contact with the patient's skin [4]. US properties to tendon recovery after sports injury, in turn, probably may be explained by upregulation of proliferating cell nuclear antigen (PCNA; *in vitro* study of the Achilles tendon in a rat) [7]. The treatment stimulates fibroblasts to collagen synthesis at the second stage of healing (proliferation phase) and provides an antiedematous “drainage” action in the case of posttraumatic articular effusions with the effects visible to the naked eye already after one session. Therapeutic US seems to have a slightly proinflammatory effect that optimizes the effectiveness of damaged tissue repair. The healing process is extremely complicated, particularly when it occurs in multiple tissue types simultaneously. It progresses at different levels and requires revascularization. In the case of partly tendon rupture or excessive stretch, a small amount of effusion, tenderness, joint instability, and discomfort during effort can occur. Tissue damage and the accompanying inflammation initiate the regeneration processes. The last stage of healing (matrix-remodelling phase) includes collagen synthesis and scar formation [8]. What is crucial in this process is the adequate response of the body systems and tissues to the injury, as an insufficient or, more frequently, excessive inflammatory reaction leads to problematic healing which results in a reduction of tissue/organ functionality [9]. An important role is also played by the oxidant-antioxidant equilibrium, as oxidants (reactive oxygen species (ROS) and/or reactive nitrogen species (RNS)) mediate virtually all relevant cell functions. Oxidants are also mediators of the response to the disturbance of homoeostasis. When the oxidant-antioxidant equilibrium is disrupted, usually in association with oxidative stress (excessive generation of ROS/RNS), a change in the properties or dysfunction of cellular biomolecules occurs due to oxidative damage, which is accompanied by, e.g., inflammation and increased activity of lysosomal enzymes [10, 11]. Among the consequences of imbalance in the oxidation/reduction processes may be DNA mutations, denaturation, or aggregation of proteins, as well as increased lipid peroxidation which, depending on the intensity or duration of the disrupting factor, leads to necrosis, apoptosis, or inflammation [11]. Many studies have proven that prolonged inflammation is accompanied by chronic oxidative stress. Therefore, a vicious circle occurs which can result in severe chronic diseases, including cancer, diabetes mellitus, neurodegenerative disorders (e.g., Alzheimer's and Parkinson's diseases), rheumatoid arthritis, and cardiovascular and lung diseases. Among other consequences of chronic inflammation and oxidative stress are atherosclerosis, hypertension, ischaemia-reperfusion injury, or increased rate of aging [11, 12]. However, necrosis, apoptosis, and inflammation are not the only types of the organism response to the elevated ROS/RNS concentrations. Instead, an increase in the antioxidant capacity can occur as a result of adaptation of the body to a short-term or low-level stimulus. Increased antioxidant capacity is observed, e.g., during adaptation to high ROS concentrations induced by physical effort [13] or in tumour cells, which can result in acquired tumour radioresistance [12]. The crucial elements of the antioxidant system are antioxidant enzymes, especially superoxide dismutase (SOD), glutathione peroxidase (GPx), and catalase (CAT) [11], while among many markers of oxidative stress lipid peroxidation products are particularly known (e.g., thiobarbituric acid reactive substances (TBARS) including malondialdehyde (MDA)) [14]. Redox balance is also related to the activities of lysosomal enzymes, since they are associated with inflammation [15]. Lysosomal enzymes are also associated with the serine protease inhibitors [16]. Accordingly, the aim of the study was to assess the effect of single DEMS and US sessions on the concentration of TBARS and the activities of SOD, GPx, and CAT, as well as the activities of selected lysosomal enzymes (acid phosphatase (AcP), arylsulfatase (ASA), and cathepsin D (CTS D)) and the activity of the serine protease inhibitor, *α*_1_-antitrypsin (AAT), in the peripheral blood of amateur athletes who had an injury in the lumbosacral segment of the spine, shoulder, or ankle joint.

## 2. Material and Methods

### 2.1. Subject

The study involved 30 amateur athletes (22 females and 8 males) subjected to DEMS procedure and 31 amateur athletes (17 females, 14 males) subjected to US procedure. The athletes attended sports clubs in Bydgoszcz, Poland (characteristics in Table 1). They practised indoor volleyball or canoeing. The participants were informed in detail about the aim and course of the experiment and signed their consent to participate in the study (parents in the case of minor ones). The participants were requested not to change their nutritional and sports-related habits throughout the study. They were examined by a sports physician at the beginning of the sports season and were permitted to continue practising their sport disciplines. The study excluded athletes with a history of surgical interventions, a viral or bacterial infection, with fever or other inflammatory sign, and those with injuries other than those considered in the study, as well as the athletes which were taking any medications (e.g., painkillers) and dietary supplements that could affect the redox balance in the organism. The study was approved by the Bioethics Committee of the Nicolaus Copernicus University in Toruń, Collegium Medicum in Bydgoszcz, Poland (consent no. KB 395/2013). The study was also registered in the public web-based clinical study database (Clinical Trial Registry-India, no. CTRI/2018/01/011344).

### 2.2. Protocol

Four groups were distinguished among the study participants—two control and two study groups (Table 1). One study group included athletes with pain in the lumbosacral segment of the spine (low back pain (LBP)) caused by disturbances in the musculofascial tonus. They were treated with one DEMS procedure. The other one included athletes with pain in the shoulder or ankle joint with features of tendinopathy. These athletes were subjected to a single session of low-frequency US. Immediately before the procedure, DEMS-treated injured athletes assessed their pain at 3.4 ± 1.5, while US-treated injured athletes at 4.2 ± 1.9 points on the visual analogue scale (VAS) (0 = no pain, 5 = moderate pain, and 10 = the most intense pain imaginable). All athletes also subjectively assessed their overall health status on a scale of 0 to 10 (0 = the worst possible, 10 = perfect). The healthy (control) individuals identified the status at 8.0 ± 1.1 (DEMS) and 9.2 ± 0.9 points (US), while the injured ones at 7.4 ± 1.4 and 6.8 ± 1.2 points, respectively. DEMS was conducted using the Salus Talent device. The following parameters of the procedure were used: magnetic induction 1.3 T (approx. 50% of maximum value, i.e., 2.5 T), electromagnetic field frequency 50 Hz (max. value), and treatment duration 20 minutes. The disc-shaped transducer emitting electromagnetic field was applied to the lumbosacral segment of the spine. US was conducted with frequency 38 Hz in continuous mode using US device and USG gel (neutral for health; without medicines). The energy supplied to the subject's skin amounted 1.5 W/cm^2^ (50% of maximum value of the device). The applied treatments were clearly felt by the participants deep in their tissues and were set to induce analgesic effect. After the sessions, the injured sportsmen evaluated their pain at 3.2 ± 1.4 (DEMS treated) and 3.8 ± 1.6 (US treated) on the VAS, whereas their health status at 7.5 ± 1.5 and 7.0 ± 1.3, respectively. The control individuals assessed their health status at 8.1 ± 1.1 after DEMS and 9.0 ± 1.1 after US.

### 2.3. Material

The study was based on laboratory analyses of venous blood taken from the basilic vein before the procedure and 30 minutes and 24 hours thereafter (2 vacuum tubes/individual: 1 with potassium versenate for blood plasma, vol. 4 mL, and 1 with coagulation activator and separation gel for serum, vol. 5 mL).

### 2.4. Methods

All biochemical methods used in the study were based on the spectrophotometric measurements of absorbance in the tested solutions.

In order to determine the concentration of TBARS in plasma and erythrocytes (TBARSpl and TBARSer, respectively), thiobarbituric acid (TBA) at 0.375%, trichloroacetic acid (TCA) at 15%, and hydrochloric acid (HCl) at 0.25 mol/L were added to tubes with plasma or erythrocyte suspension (50% *v*/*v* in PBS). The mixtures were incubated for 20 min at 100°C, cooled down to 4°C, and centrifuged for 15 min. Subsequently, supernatant was collected, and its absorbance at a wavelength of 532 nm was measured [17, 18]. Moreover, the activities of superoxide dismutase (SOD; EC 1.15.1.1), glutathione peroxidase (GPx; EC 1.11.1.9), and catalase (CAT; EC 1.11.1.6) were measured in the erythrocyte suspension. The method of determination of the SOD activity involved measuring the absorbance of a mixture of erythrocyte suspension and adrenaline at pH > 7. In such conditions, the enzyme inhibits adrenaline autoxidation to adrenochrome, as manifested by the change in the absorbance of the solution which corresponds to the activity of the enzyme [19]. Similarly, the erythrocyte activity of GPx was measured. Absorbance was measured after the reaction that occurred in the solution between the enzyme, hydrogen peroxide, and glutathione, a cofactor in the reaction [20]. The CAT activity was determined in a similar way. Changes in the absorbance of mixtures of hydrogen peroxide with the tested erythrocyte suspensions were measured [21].

In the peripheral blood serum of the study participants, the activity of the following enzymes was determined: AcP (EC 3.1.3.2), ASA (EC 3.1.6.1), CTS D (EC 3.4.23.5), and AAT. The measure of the AcP activity was the amount of p-nitrophenol generated during hydrolytic decomposition of p-nitrophenylphosphate. The reaction was buffered using 0.5 mol/L citrate-tartrate-formaldehyde buffer at pH 4.9. Absorbance was measured at a wavelength of 405 nm [22]. The ASA activity was determined by adding 0.01 mol/L 4-nitrocatechol sulphate and 0.5 mol/L acetate buffer at pH 5.6 to the tested serum. 4-Nitrocatechol sulphate was decomposed by the enzyme to 4-nitrocatechol. Absorbance was measured at a wavelength of 510 nm [23]. The CTS D activity measurement was based on the hydrolysis of 2% denatured bovine haemoglobin by the enzyme present in the tested serum at 37°C. The reaction was terminated using an aqueous solution of sodium hydroxide (NaOH) at 0.1 mol/L, and a phenolic reagent was added which bound the hydrolysed haemoglobin and stained the solution blue. Absorbance was measured at a wavelength of 660 nm [24]. The AAT activity was directly proportional to the reduction of the enzymatic activity of trypsin after incubation with the tested blood serum (10 min, 25°C). The reaction was inhibited using 30% acetic acid, and the absorbance of the solution was measured at a wavelength of 410 nm. Finally, the AAT activity was read from a calibration curve generated using soybean trypsin inhibitor [25].

### 2.5. Statistical Analysis

Compatibility with the normal distribution was analysed using the Kolmogorov–Smirnov test, while the homogeneity of variances was established using Levene's test. The main analysis was based on the ANOVA test with Tukey's HSD (honestly significant difference) test for different *N* as post hoc analysis. If the results did not meet the criteria for parametric tests, the Kruskal–Wallis test with multiple comparisons was performed. Additionally, Pearson's correlation coefficients were determined. The results are shown as arithmetic means ± standard deviations. Differences between the means were considered as statistically significant at *p* < 0.05.

## 3. Results

As regards DEMS, the greatest difference was demonstrated in the activity of CTS D in the injured (LBP) athletes 24 hours after the treatment in relation to the baseline assay, before the session. The activity was over 2 times lower after the treatment (54.5% difference, *p* = 0.052, Table 2). The enzyme activity in these athletes was also clearly (23.2%) lower 30 minutes after DEMS compared to the baseline (*p* > 0.05). The same trend in the changes of the CTS D activity was also observed in the other study groups, and after the US treatment, the enzyme activity in the injured athletes was also the lowest at the 24-hour study time point (52.1% difference, *p* = 0.086). However, in the US-treated healthy controls, the decrease of the CTS D activity after the session was statistically significant, both at the 30 min (*p* < 0.05) and 24 h time points (*p* < 0.01) (47.5% and 55.7% differences, respectively), whereas in the control athletes after DEMS it was statistically insignificant. There were also no statistically significant differences in the CTS D activity between the control and injured athletes in both procedures, as well as between the procedures.

In all study groups, exactly the same character in the changes of SOD activity after the treatment was also noted. However, the differences were statistically insignificant, similarly as regards the other parameters. The SOD activity was higher 30 minutes after the given treatment compared to the baseline, but 24 hours thereafter practically returned to the initial state (before the treatment). It can also be stated that the CAT activity was slightly greater after the treatments in relation to the baseline assays (30 min and/or 24 h after them); however, it is difficult to find any other tendencies. The levels of other parameters changed in different ways.

**Figure 1:**
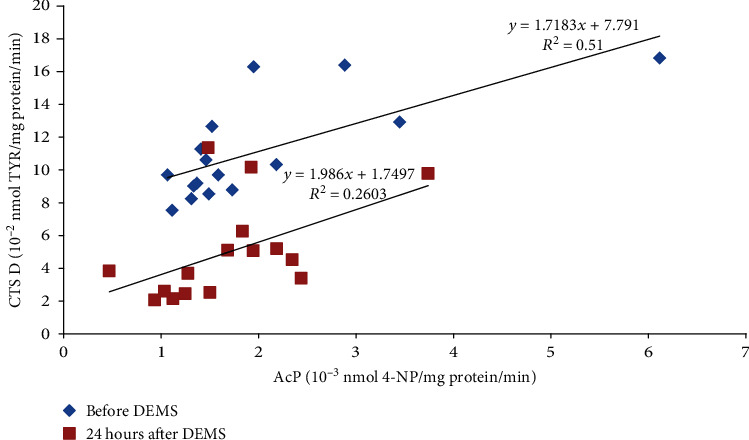
Positive linear correlations between the serum AcP and CTS D activities before and 24 h after the DEMS procedure in the injured sportsmen: *r* = 0.71 (*p* < 0.01) and *r* = 0.51 (*p* < 0.05), respectively. DEMS: deep electromagnetic stimulation; 4-NP: 4-nitrophenol; TYR: tyrosine.

**Figure 2:**
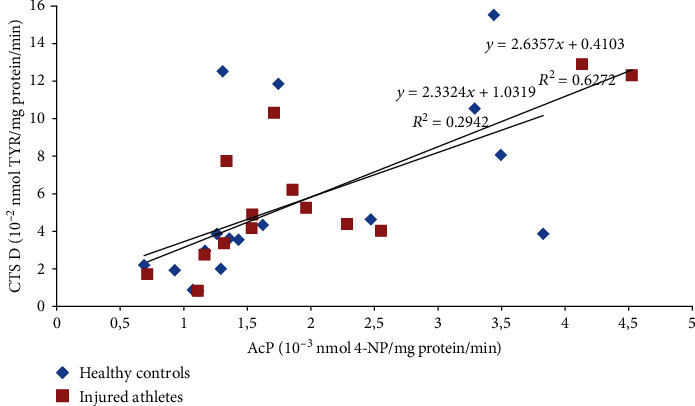
Linear regressions of the serum AcP and CTS D activities 24 h after the US procedure in the healthy and injured sportsmen (*r* = 0.54, *p* < 0.05, and *r* = 0.79, *p* = 0.001, respectively). US: ultrasound; 4-NP: 4-nitrophenol; TYR: tyrosine.

**Figure 3:**
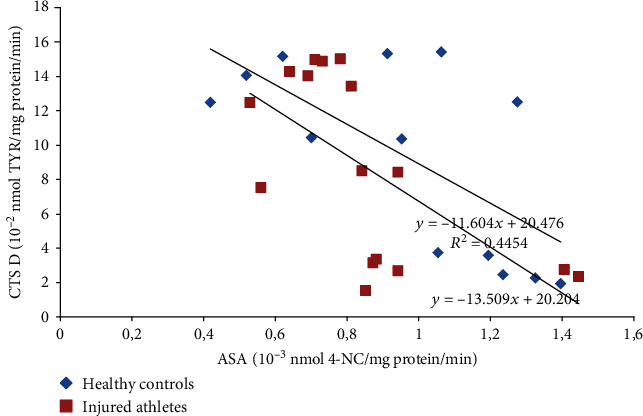
Negative linear correlations between the serum ASA and CTS D activities 30 min after the single DEMS procedure in the healthy and injured sportsmen (*r* = −0.67, *p* < 0.05, and *r* = −0.63, *p* < 0.01, respectively). DEMS: deep electromagnetic stimulation; ASA: arylsulfatase; 4-NC: 4-nitrocatechol; CTS D: cathepsin D; TYR: tyrosine.

**Figure 4:**
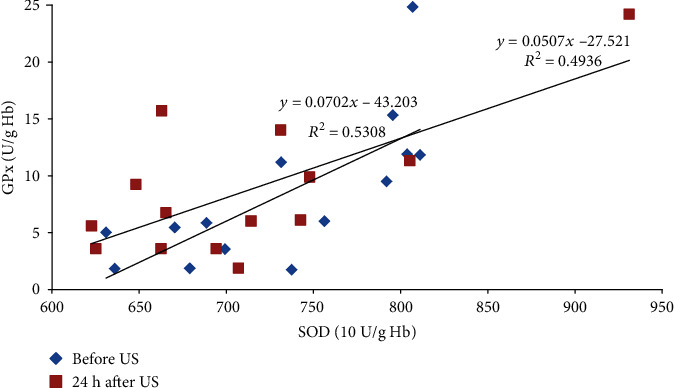
Linear relationships between the erythrocyte SOD and GPx activities before and 24 h after US in the injured athletes (*r* = 0.73 and *r* = 0.70, *p* < 0.01, respectively). US: ultrasound; SOD: superoxide dismutase; GPx: glutathione peroxidase; Hb: haemoglobin.

Moreover, the study revealed many statistically significant linear relationships between the parameters. Before the DEMS session and 24 h after it, the AcP activity in the LBP athletes positively correlated with the CTS D activity (*r* = 0.71 and *p* < 0.01 and *r* = 0.51 and *p* < 0.05, respectively; Figure 1). Similar linear relationship was also found 24 h after US, both in the healthy control and injured athletes (*r* = 0.54 and *p* < 0.05 and *r* = 0.79 and *p* = 0.001, respectively; Figure 2). The ASA activity measured 30 minutes after DEMS was negatively correlated with the CTS D activity in the noninjured and injured athletes (*r* = −0.67 and *p* < 0.05 and *r* = −0.63 and *p* < 0.01, respectively; Figure 3) and positively before US in the healthy controls (*r* = 0.75, *p* < 0.001). In turn, 24 hours after the US treatment in the healthy controls, the ASA activity negatively correlated with the AAT activity (*r* = −0.49, *p* < 0.05). 30 minutes after US, a positive linear correlation between the TBARSer concentration and the GPx activity was also revealed in the injured athletes (*r* = 0.63, *p* < 0.05), similarly as the SOD and GPx activities before and 24 h after the procedure (*r* = 0.73 and *r* = 0.70, *p* < 0.01; Figure 4), whereas at 24 h time point in the US-treated healthy controls the TBARSer concentration was negatively correlated with the SOD activity (*r* = −0.55, *p* < 0.05). Furthermore, a number of statistically significant Pearson's linear correlations between redox balance parameters and lysosomal enzymes (Table 3) as well as between the AAT and GPx activities 30 min after DEMS (*r* = −0.89, *p* < 0.001) and between the AAT activity and the TBARSer concentration 24 h after US (*r* = −0.63, *p* < 0.01) in the control participants were also found.

## 4. Discussion

The study showed single DEMS and US sessions did not disturb the oxidant-antioxidant equilibrium in the healthy and injured juvenile amateur sportsmen, since no statistically significant differences in measurements of the TBARS concentration and antioxidant enzyme activities (SOD, GPx, and CAT) were demonstrated. The TBARSpl level tended to increase after the procedures in the groups of control and injured athletes, while there was no clear trend in the changes of TBARSer concentration. The SOD activity changed as a result of the treatment in a similar manner in both procedures (*p* > 0.05). TBARS are mainly malondialdehyde (MDA), which belongs to the specific markers of oxidative stress as a secondary product of lipoperoxidation induced by free radicals (nonenzymatic lipid peroxidation) [14]. The antioxidant enzymes analysed in the study are also reliable indicators for the evaluation of ratio between the oxidation and reduction reactions. These enzymes “scavenge” ROS and RNS: superoxide anion (O_2_^·–^) (SOD), as well as hydrogen peroxide (H_2_O_2_) and organic peroxides (GPx and CAT) [26]. CAT demonstrates peroxidase activity at low concentration of H_2_O_2_ and oxidizes ethanol, methanol, phenols, formates or nitrites, and mentioned organic peroxides. The role of an H_2_O_2_ scavenger is then played almost completely by GPx [27]. The enzymes prevent the generation of other dangerous free radicals as well—primarily the hydroxyl radical (HO^·^) and peroxynitrite (ONOO^–^) [11]. Moreover, linear relationships found in the US procedure in the context of no differences between the results suggest rather the proper functioning of the antioxidant barrier or at most slightly increased production of ROS. 30 min after the treatment, strong positive linear correlations between the TBARSer concentration and the GPx activity (*r* = 0.63, *p* < 0.05) as well as between the SOD and GPx activities before and 24 h after the procedure (*r* = 0.73 and *r* = 0.70, *p* < 0.01; Figure 4) were shown in the injured athletes, whereas at 24 h study time point in the US healthy controls the TBARSer concentration was negatively correlated with the SOD activity (*r* = −0.55, *p* < 0.05), which can suggest that the likely increase in ROS production was rather a result of the injury, not the procedure.

The study also showed practically no impact on the lysosomal balance. Basically, there were no statistically significant changes in the activity of lysosomal enzymes. It seems only that the US session and probably also DEMS decrease lysosomal proteolytic activity in the blood serum, since the main proteolytic enzyme in lysosomes is CTS D [28], and its activity was meaningfully lower after the procedures both in the noninjured and injured sportsmen (reference time point—the baseline assay). After US, it was statistically significant difference. Lysosomal enzymes are acid hydrolases. Thanks to their ability to decompose biomolecules and their abundance (more than 60 types), cells are able to regulate many different processes, such as apoptosis, necrosis, phagocytosis, exocytosis, or removal of waste and dysfunctional proteins [15, 29]. Due to the aforementioned, lysosomes are also involved in the positive and negative regulation of inflammation [30]. Increased permeability of lysosomal membranes and thus higher activity of lysosomal hydrolases in the cytosol and/or intercellular space and blood plasma/serum can occur in response to damaging factors (biological, chemical, and physical) based on the free radical mechanism. ROS/RNS can oxidize the protein-lipid lysosomal membrane and increase its permeability, as in the case of other biological membranes, including the cell membrane [15, 31]. Lysosomal enzymes can also be a source of ROS/RNS *per se*, e.g., AcP in Fenton reaction due to the presence of Fe^2+/3+^ in the active site. In such a way, overactive AcP might carry out the fragmentation of collagen within osteoclasts in skeletal disorders involving the excessive activity of these cells. In this type of disorders, increased AcP activity has been shown in the patients' blood serum [32]. The enzyme can also be overproduced by macrophages and dendritic cells in the course of cancer [33] and rheumatoid arthritis [34], and its free radical activity occurs in macrophages during antigen presentation [35]; however, the basic function of this enzyme is removal of a phosphate group from phosphoric (V) acid monoesters [34]. Overexpression and the presence in peripheral blood have also been observed in the case of CTS D during tumour progression, similarly in the course of Alzheimer's disease. In general, high activity of the enzyme appears in damaged tissues and sites of intense inflammation [28]. It has also been proven that this protein is released from lysosomes into the cytoplasm during apoptosis induced by oxidative stress [15]. In turn, mutations in the ASA gene resulting in a total loss or severe reduction of the enzyme activity can lead to neurodegenerative disorders [36]. It has been demonstrated that a single dose of human ASA administered into the damaged spinal cord in mice causes a significant improvement of their locomotor function [37]. ASA hydrolyses esters of sulfuric acid, which is important in, e.g., the processing of glycosphingolipids of the myelin sheath of neurons [36]. In the sportsmen's blood serum, in both procedures of the study, the character in changes of the CTS D and AcP activities was similar (Figures 1 and 2). However, relationships between the CTS D and ASA activities were opposite, comparing the DEMS and US treatments. The ASA activity measured 30 min after DEMS was negatively correlated with the CTS D activity in the injured and noninjured athletes (Figure 3) and positively before US in the healthy controls (*r* = 0.75, *p* < 0.001). Possibly, it was caused by selective release of enzymes from lysosomes due to their random aggregation after pH increase inside the lysosome [38]. The positive relationship described here between ROS and lysosomal activity is confirmed by numerous statistically significant linear correlations obtained in the study (Table 3). Thus, these proper relations (correlations) between the parameters may confirm the maintenance of the redox and lysosomal equilibriums in the healthy and injured subjects.

Differences in the serum activity of AAT in response to single DEMS and US procedures were statistically insignificant as well. AAT is a sialoglycoprotein, which belongs to *ser*ine *p*rotease *in*hibitor*s* (SERPINS) and is classified as a positive acute-phase protein (APP), along with C-reactive protein, fibrinogen, and *α*_1_-acid glycoprotein, because its activity in blood increases in response to damage and inflammation [16]. Therefore, many APPs are considered as classic biomarkers of inflammation (especially C-reactive protein, fibrinogen, and complement factors) [9]. The main function of these proteins is to protect the organism against further damages, help to restore the homoeostasis, and adapt to the harmful stimulus. AAT as a serpin inhibits neutrophilic proteases: elastase, cathepsin G, and proteinase 3. It is secreted mainly by hepatocytes, but can also be abundantly produced by macrophages, as well as intestinal and bronchial epithelial cells. Moreover, it has been discovered that AAT inhibits aggregation of certain proteins (e.g., alcohol dehydrogenase, aldolase, carbonic anhydrase, and catalase) induced by high temperature or chemical compounds; hence, AAT is also considered as an extracellular chaperone [16]. It has been also demonstrated in this study that the activity of AAT can negatively correlate in healthy men's blood serum with the ASA activity (the US-treated healthy controls, 24 h after the procedure: *r* = −0.49, *p* < 0.05), which confirms again no apparent disturbance of lysosomal balance. Similarly, correlations between AAT and parameters of oxidant-antioxidant balance in the healthy controls suggest no disturbances in the balance (AAT vs. GPx 30 min after DEMS: *r* = −0.89, *p* < 0.001; AAT vs. TBARSer 24 h after US: *r* = −0.63, *p* < 0.01).

In conclusion, no significant differences were demonstrated comparing the healthy and injured athletes, as well as both procedures. In general, the observed trends in the changes of the analysed parameters in the study were also similar in nature in both procedures. Therefore, the obtained results suggest that the conducted DEMS and US sessions did not have significant effect on the described physiological functions, except for lysosomal proteolytic activity in blood serum.

## 5. Conclusions

Single sessions of deep electromagnetic stimulation and low-frequency ultrasound do not significantly disturb the oxidant-antioxidant and lysosomal equilibriums, nor do they have impact on acute-phase response of the *α*_1_-antitrypsin in the venous blood of amateur juvenile athletes. However, it seems that the US treatment significantly decreases proteolytic activity of cathepsin D in the healthy athletes' blood serum, as well as probably also in the injured athletes and after the DEMS treatment.

## Figures and Tables

**Table 1:** Characteristics of the study participants (mean ± SD).

	DEMS_healthy controls_	DEMS_injured athletes_	US_healthy controls_	US_injured athletes_
Number	14	16	17	14
Age (years)	17.9 ± 5.6	16.4 ± 2.6	22.3 ± 8.3	16.8 ± 4.0
Body mass (kg)	69.8 ± 12.1	61.8 ± 7.3	67.5 ± 13.2	69.6 ± 13.6
Body height (m)	1.8 ± 0.1	1.7 ± 0.1	1.7 ± 0.07	1.8 ± 0.1
Body mass index (kg/m^2^)	21.5 ± 3.4	21.4 ± 2.1	23.3 ± 3.3	21.5 ± 2.5
Training experience (years)	4.5	4.4	2.2	3.8

**Table 2:** Oxidative stress parameters together with lysosomal and antiprotease activities in the peripheral blood of juvenile athletes subjected to single DEMS and low-frequency ultrasound (US) sessions.

	DEMS_healthy controls_ (*N* = 14)			DEMS_injured athletes_ (*N* = 16)			US_healthy controls_ (*N* = 18)			US_injured athletes_ (*N* = 14)		
	I	II	III	I	II	III	I	II	III	I	II	III
TBARSpl	4.0 ± 0.6	4.1 ± 0.4	4.2 ± 0.8	4.2 ± 0.8	4.2 ± 0.7	4.5 ± 0.7	4.1 ± 0.6	3.9 ± 0.6	4.2 ± 0.7	3.8 ± 0.9	3.6 ± 0.5	4.0 ± 0.6
TBARSer	23.5 ± 6.1	23.7 ± 7.9	23.9 ± 8.0	21.5 ± 5.8	21.8 ± 6.1	21.3 ± 3.3	24.1 ± 8.0	21.8 ± 5.0	26.8 ± 12.3	24.3 ± 4.0	27.8 ± 9.8	24.6 ± 11.0
SOD	77.3 ± 9.8	81.4 ± 12.6	77.1 ± 9.7	74.0 ± 12.7	78.0 ± 10.2	76.5 ± 13.1	69.6 ± 6.3	71.0 ± 4.0	67.3 ± 3.2	73.1 ± 6.5	76.8 ± 9.6	71.1 ± 8.2
GPx	7.0 ± 5.4	7.1 ± 5.2	8.8 ± 3.6	8.6 ± 7.1	7.2 ± 3.7	7.0 ± 4.6	9.3 ± 7.9	5.6 ± 5.0	8.0 ± 5.3	8.2 ± 6.3	7.5 ± 5.6	8.5 ± 5.9
CAT	60.8 ± 7.3	60.5 ± 6.8	62.3 ± 10.6	59.5 ± 8.9	60.6 ± 9.7	61.9 ± 11.6	59.9 ± 6.8	59.7 ± 4.7	64.1 ± 8.6	61.2 ± 10.9	58.5 ± 9.0	61.5 ± 10.1
AcP	1.6 ± 0.5	1.6 ± 0.7	1.4 ± 0.5	2.0 ± 1.2	1.9 ± 1.0	1.7 ± 0.8	1.8 ± 0.8	1.7 ± 0.9	1.9 ± 1.0	2.1 ± 1.0	2.1 ± 0.9	2.0 ± 1.1
ASA	0.8 ± 0.3	1.0 ± 0.3	1.0 ± 0.4	0.7 ± 0.2	0.9 ± 0.2	0.8 ± 0.3	0.9 ± 0.3	0.8 ± 0.2	0.8 ± 0.2	0.7 ± 0.2	0.7 ± 0.1	0.7 ± 0.2
CTS D	10.5 ± 2.0	9.1 ± 5.5	7.7 ± 4.2	11.2 ± 0.3	8.6 ± 5.4	5.1 ± 3.0	12.2 ± 3.8	6.4 ± 4.1^∗^	5.4 ± 4.4^∗∗^	11.7 ± 3.1	8.3 ± 5.2	5.6 ± 3.7
AAT	8.3 ± 1.4	7.7 ± 1.3	8.3 ± 1.4	8.9 ± 1.8	8.2 ± 1.4	8.7 ± 1.0	7.8 ± 1.4	7.7 ± 1.2	7.6 ± 1.0	7.8 ± 1.6	7.3 ± 1.4	8.1 ± 1.2

Values are means ± SD. Statistically significant differences were revealed in relation to measurement I in the US-treated healthy controls (^∗^*p* < 0.05, ^∗∗^*p* < 0.01). Measurement I: before the treatment; measurement II: 30 min after the treatment; measurement III: 24 hours after the treatment; TBARSpl/er: thiobarbituric acid reactive substances in plasma (10^–1^ nmol MDA/mL plasma)/erythrocytes (nmol MDA/g Hb); MDA: malondialdehyde; Hb: haemoglobin; SOD: superoxide dismutase (10 U/g Hb); GPx: glutathione peroxidase (U/g Hb); CAT: catalase (IU/g Hb); AcP: acid phosphatase (10^–3^ nmol 4-NP/mg protein/min); 4-NP: 4-nitrophenol; ASA: arylsulfatase (10^–3^ nmol 4-NC/mg protein/min); 4-NC: 4-nitrocatechol; CTS D: cathepsin D (10^–2^ nmol TYR/mg protein/min); TYR: tyrosine; AAT: *α*_1_-antitrypsin (10^–1^ mg TR/mL serum); TR: trypsin.

**Table 3:** Pearson's linear correlations between parameters of redox balance and lysosomal enzymes.

Before DEMS in the injured athletes	SOD vs. CTS D	*r* = 0.55, *p* < 0.05
24 h after DEMS in the injured athletes	SOD vs. ASA	*r* = 0.58, *p* < 0.05
Before US in the controls	TBARSer vs. ASA	*r* = 0.58, *p* < 0.05
30 min after US in the controls	CAT vs. CTS D	*r* = 0.49, *p* < 0.05
24 h after US in the controls	TBARSer vs. ASA SOD vs. CTS D CAT vs. CTS D	*r* = 0.51, *p* < 0.05 *r* = 0.62, *p* < 0.01 *r* = 0.66, *p* < 0.01
Before US in the injured athletes	SOD vs. AcP GPx vs. AcP	*r* = 0.59, *p* < 0.05 *r* = 0.65, *p* < 0.05
30 min after US in the injured athletes	GPx vs. AcP	*r* = 0.66, *p* < 0.05
24 h after US in the injured athletes	TBARSer vs. ASA	*r* = 0.65, *p* < 0.05

DEMS: deep electromagnetic stimulation; SOD: superoxide dismutase; CTS D: cathepsin D; ASA: arylsulfatase; US: ultrasound; TBARSer: thiobarbituric acid reactive substances in erythrocytes; CAT: catalase; AcP: acid phosphatase; GPx: glutathione peroxidase.

## Data Availability

The data used to support the findings of this study are included within the article.
